# Kinetic Models for the Changes in Moisture, Bioactive Compounds, and Antioxidant Activities During the Storage of Tu Quy Mango Powder (*Mangifera indica* L.)

**DOI:** 10.1002/fsn3.70509

**Published:** 2025-06-27

**Authors:** Ngoc Duc Vu, Trinh Thi Nhu Hang Nguyen, Thi Hoang Nhi Dang, Binh An Pham

**Affiliations:** ^1^ Institute of Applied Technology and Sustainable Development Nguyen Tat Thanh University Ho Chi Minh City Vietnam; ^2^ Center for Hi‐Tech Development Nguyen Tat Thanh University, Saigon Hi‐Tech Park Ho Chi Minh City Vietnam; ^3^ Faculty of Chemical Engineering and Food Technology Nong Lam University Ho Chi Minh City Vietnam

**Keywords:** freeze‐dried mango, kinetics, powder, shelf life

## Abstract

Freeze‐dried mango powder is prone to quality degradation during storage. Variations in raw material quality and production conditions across batches further complicate long‐term quality control. Repeated and lengthy shelf life testing is both time‐consuming and costly. Therefore, mathematical models play a crucial role in estimating shelf life, reducing costs, and tailoring storage conditions for each production batch. The objective of this study was to develop and propose mathematical models to quantitatively predict changes in moisture content, bioactive compounds, and antioxidant activity in mango powder over time. Mango powder was stored at four different temperatures (20°C–55°C) corresponding to RH = 33%–55%. Mathematical models were developed based on statistical parameters (*R* ^2^, RMSE, *χ* ^2^). The results showed that moisture content was dependent on storage temperature, with the most suitable model being Vu (*R* ^2^ > 0.97) at all four temperatures. Zero‐order, first‐order, and Weibull models were considered suitable to describe the mechanisms of bioactive components and antioxidant activity with *R* ^2^ > 0.99. The models predicted the shelf life of the product from 85 to 551 days depending on the storage conditions. Additionally, the equilibrium moisture content of mango powder under the current storage conditions was found to be less than 5%. The study's results provide experimental models and form the basis for improving product quality, predicting shelf life, and establishing automated sample control in production.

## Graphical abstract

Freeze‐dried mango powder retains the characteristics of fresh fruit, offering health benefits, but its quality during storage is influenced by temperature and humidity. This study developed mathematical models to describe moisture absorption, bioactive compound degradation, and antioxidant activity, helping predict the shelf life of mango powder under different storage conditions. The results showed that the Vu model best predicted moisture content, whereas other models effectively described the degradation of bioactive compounds and antioxidant activity, aiding in quality control and shelf life estimation.

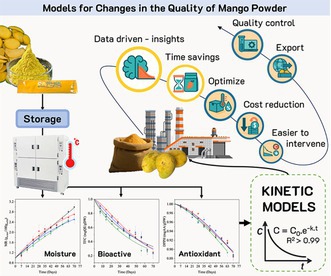

## 1 Introduction

Mango ( *Mangifera indica* L.) is one of the most nutrient‐dense fruits. Per 100 g of fresh fruit, it contains approximately 15 g of carbohydrates, 0.38 g of fat, 0.82 g of protein, and provides about 60–190 kcal (Jalali et al. 2022; Yahia et al. 2023). Additionally, several prominent amino acids, such as lysine, leucine, valine, threonine, isoleucine, and histidine (0.01–0.066 g/100 g) are found (Yahia et al. 2023). Some other components like thiamine, riboflavin, niacin, and minerals, with concentrations ranging from 0.01 to 1.31 mg/100 g, particularly bioactive compounds, such as vitamin C, phenolics, and flavonoids, which are present in high amounts (9–186 mg/100 g) (Sultana et al. 2012; Thao et al. 2023). These nutrients enhance vision, immune function, digestion, prevent cancer, slow aging, and improve cholesterol and blood lipid levels (Jalali et al. 2022; Yahia et al. 2023; Zeng et al. 2020). Therefore, technologies need to be applied to enhance the ability to maintain quality and extend the shelf life of the product.

Freeze‐drying is one of the technologies that helps preserve most of the nutritional components during the drying process. However, improper storage conditions can lead to rapid degradation of the nutrients, reducing the product's value (Trang et al. 2023). Indeed, about 55% of the polyphenols have been reported to degrade during the storage process of soursop tea (Do et al. 2024). Similarly, more than 40% of vitamin A in powdered enteral has been degraded during storage at 35°C (Yang et al. 2022). Therefore, several previous reports have developed kinetic models and applications to predict and control the quality of certain fruit powders, such as soursop, shallot, and melon. The findings have indicated that soursop powder exhibited color changes following a zero‐order model (Chang et al. 2018), whereas shallot powder's quality indices, with shelf life estimated between 245.64 and 447.37 days, followed a similar model (Sukasih et al. 2019). Coconut powder exhibited bioactive and antioxidant changes following zero, first, and second‐order models (Lucas‐Aguirre et al. 2020). Melon powder, processed by spray drying, followed first‐order model for changes in physical properties and carotenoid content during 180 days of storage (Tan et al. 2021).

Alternatively, thanks to freeze‐drying technology, mango powder product retained the natural mango flavor and effectively preserved the components present in the raw material (Mawilai 2019). However, the quality of raw materials in each batch as well as production and storage conditions significantly affects the shelf life of the product (Vu, Pham, et al. 2025). Long‐term, repeated testing to accurately determine the shelf life of each production batch incurs high costs and time. Mathematical models offer a solution to reduce investigation costs and time for businesses (Trang et al. 2023). Indeed, these models allow for quick shelf life estimation under storage conditions without the need for prolonged waiting, and they make it easier to adjust storage conditions to meet quality goals. Additionally, models integrated into automated monitoring systems can enhance quality control during storage. However, in‐depth reports on the mechanisms of changes, model building, and predictions of shelf life of freeze‐dried mango powder are still limited. In particular, no comprehensive studies comparing multiple quality indices from a single experiment have been reported. A thorough understanding of appropriate storage conditions will help control quality, extend the product's shelf life, and enhance its commercialization potential in international markets.

Therefore, the objective of this study is to simultaneously explore the mechanisms of changes in bioactive compounds and antioxidant activity under different storage conditions in order to provide an overview of quality changes and comparisons. Experimental data were used to calculate statistical parameters, build mathematical models to describe the mechanisms of changes in the components, and determine the shelf life of freeze‐dried Tu Quy mango powder. The research results serve as a scientific basis for practical application in the production process effectively, conveniently for each production stage, and provide a foundation to promote automation in quality control.

## 2 Materials and Methods

### 2.1 Materials

Tu Quy mango was harvested in Ben Tre province, Vietnam (10°14′54″ N and 106°22′34″ E). The criteria for selecting raw materials included a weight of 1.00 ± 0.2 kg and ripeness at the time when total soluble solids reached 16° ± 2° Bx. The mango flesh was pureed and heat‐treated by water bath at a product core temperature of 85°C ± 2°C for 10 min. The puree mixture was rapidly cooled with cold water (5°C ± 2°C). Citric acid was used to adjust the pH to 3.2, aiming to enhance the inactivation of polyphenol oxidase. The puree mixture was blended with maltodextrin (DE = 10) in a ratio of puree (85%): maltodextrin (15%) and shaped in drying trays (50 × 50 × 1 cm). The freezing process was carried out at −20°C for 600 min. The total duration of the freeze‐drying process lasted 36 h at a pressure of 25 Pa. After the drying process, the product was ground (YB—2000A, Yunbang, China) into fine powder, with an additive mixture, including citric acid (0.438%), flavor (5.080%), K‐sugar (0.674%), vitamin C (4.570%), and sucrose (4.498%) for 2 min to achieve a particle size of approximately 0.01 mm.

### 2.2 Chemicals and Reagents

Folin–ciocalteu (reagents), sodium carbonate 7.5% (≥ 99.8%), aluminum chloride (> 97%), potassium acetate (98.15%), ascorbic acid (≥ 99.7%), 2,2′‐Azino‐bis‐3‐ethylbenzothiazoline‐6‐sulfonic acid (ABTS) (≥ 98%), 2,2‐diphenyl‐1‐1 picrylhydrazyl (DPPH) (≥ 97%), formaldehyde (> 99.8%), sulforic acid (≥ 95%–97%), potassium iodate (> 99.8%), starch soluble (> 99%), and potassium iodide (> 99.5%), ethanol (96°) was purchased in Sigma Aldrich.

### 2.3 Procerdure

A total of 5 kg of mango powder was used in this study. Approximately 5 kg of mango powder was evenly distributed into 313 polyethylene/aluminum bags to monitor the product quality over a 70‐day storage period (PE thickness: 0.1 mm, Al thickness: 0.1 mm, length: 150 mm, width: 30 mm, water vapor transmission rate ≤ 0.5 g/[m^2^ × 24 h], oxygen transmission rate ≤ 0.5 cm^3^/[m^2^ × 24 h × 0.1 MPa]), with each bag containing 15 ± 1 g of powder. Subsequently, all the mango powder bags were evenly distributed into storage cabinets at different temperature conditions (20°C, 35°C, 45°C, and 55°C), corresponding to relative humidity values of 54%, 33%, 38%, and 45%, respectively. The temperatures selected for the investigation were based on the diversity of real‐world storage conditions, including cool room temperature (~20°C), natural ambient temperatures in several tropical countries (< 45°C), and more extreme conditions found in countries such as Ghana, the United States, and Laos (~45°C) (Sornsomboonsuk et al. 2019). In addition, the temperatures of 45°C–55°C represent accelerated conditions, serving as a basis for rapid prediction of the product's maximum shelf life (Thuy et al. 2024).

### 2.4 Analysis Methods

#### 2.4.1 Moisture Content

The heat‐resistant weighing equipment (Dragon Equipment, Ho Chi Minh City, Vietnam) continuously weighed the samples stored in storage cabinets at different temperature levels of 20°C, 35°C, 45°C, and 55°C. The moisture content of the mango powder was monitored and evaluated after 7 days using a heat‐resistant camera (M500; Xiaomi, China) (Vu et al. 2024).

#### 2.4.2 Total Flavonoid Content

The total flavonoid content was determined based on the reaction of flavonoids with aluminum chloride (AlCl_3_) to form a yellow complex [Flavonoid‐O‐Al]^+^. The color intensity is directly proportional to the flavonoid content in the sample. For each 0.5 mL of extract, 4.3 mL of ethanol, 0.1 mL of 10% aluminum chloride, and 0.1 mL of 1 M potassium acetate were mixed for the reaction over 30 min. Afterward, the total flavonoid content was determined at a wavelength of 415 nm using a UV–Vis spectrophotometer (GENESYS 10S; Thermo Scientific, USA) (Vu et al. 2023).

#### 2.4.3 Total Ascorbic Acid

Total ascorbic acid was determined based on the oxidation reaction of ascorbic acid by triiodide ions to form dehydroascorbic acid. A drop of excess iodine indicator reacts with starch solution to produce a blue‐black complex. Each 5 g of potassium iodide, 0.268 g of potassium iodate, 30 mL of 3 M sulfuric acid, and 500 mL of distilled water were mixed to obtain an iodine solution. The starch indicator was prepared with a concentration of 1 g/100 mL of hot water and kept stable for 10 min, then cooled to 35°C ± 2°C. About 1 mL of formaldehyde was used to preserve the starch solution. Each 25 mL of the extract and 1 mL of starch indicator were titrated with the iodine solution until a stable blue color appeared within 20 s. The titration of the standard Ascorbic acid was performed similarly, replacing the sample solution with a standard Ascorbic acid solution (Mohammed and Yousef 2024).

#### 2.4.4 ABTS and DPPH Antioxidant Activities

Antioxidant activity was performed based on the report by Vu et al. (2024). Each 0.5 mL of the extract was supplemented with 1.5 mL of ABTS^+^ or DPPH^•^ solution, and the reaction was carried out for 30 min. After that, the ABTS or DPPH antioxidant activity was determined at wavelengths of 734 nm (ABTS) and 517 nm (DPPH) with the assistance of a UV–Vis spectrophotometer (GENESYS 10S; Thermo Scientific, USA).

#### 2.4.5 Mathematical Model and Kinetic Analysis of the Process

Among the various moisture absorption models proposed, the most commonly used model is chosen for its mathematical simplicity and practical applicability. Notable models include the first‐order model (Equation 1) (Yan et al. 2008), the Peleg model (Equation 2) (Niamnuy et al. 2008; Thuy et al. 2024), the Weibull model (Equation 3) (Mahajan et al. 2008), and the Vu model (Equation 4), which are widely used to describe moisture absorption during the storage of dried products. These models are represented as follows:

(1) $${\mathrm{MR}}_{t}={\mathrm{MR}}_{0}\times \exp\;\left(-k_{1}\times t\right)$$

(2) $${\mathrm{MR}}_{t}={\mathrm{MC}}_{0}+\left[t/\left(k_{1}+k_{2}\times t\right)\right]$$

(3) $${\mathrm{MR}}_{t}={\mathrm{MR}}_{0}+\left({\mathrm{MR}}_{\infty }-{\mathrm{MR}}_{0}\right)\times \left[1-\exp\;\left(-t/\beta \right)\right]$$

(4) $$\begin{array}{c} {\mathrm{MR}}_{t}={\mathrm{MR}}_{0}+\left\{\left[\left(k_{1}\times a_{w}n_{1}+k_{2}\times a_{w}n_{2}-C_{0}\right)/{\mathrm{MC}}_{0}\right]-{\mathrm{MR}}_{0}\right\}\\ \;\times \left[1-\exp\;\left(-t/\beta \right)\right] \end{array}$$

A total of three commonly used kinetic models are often employed to describe changes in bioactive compounds and antioxidant capacity during storage. These include the zero‐order model (Equation 5) (Chen et al. 2020; Zheng and Lu 2011), the first‐order model (Equation 6) (Odriozola‐Serrano et al. 2009; Zheng and Lu 2011), and the Weibull model (Equation 7) (Odriozola‐Serrano et al. 2009).

(5) $$C_{t}=C_{0}-k_{1}\times t$$

(6) $$C_{t}=C_{0}\times \exp\;\left(-k_{1}\times t\right)$$

(7) $$C_{t}=C_{0}\times \exp\;\left[-{\left(k_{1}\times t\right)}^{n}\right]$$

where *k*_1_ and *k*_2_ are the reaction rate constants (day^−1^); *t* is the storage time (days); MR_*t*_ and MR_0_ are the moisture content ratios at time *t* and *t* = 0, respectively; *C*_*t*_ and *C*_0_ are the component content ratios at time *t* and *t* = 0, respectively; MR_∞_ is the equilibrium moisture content; *a*_w_ is the water activity; and *n*, *n*_1_, *n*_2_, and *β* are the kinetic parameters of the model.

#### 2.4.6 Determine the Coefficient of Determination ( *R* ^2^ ) and Chi‐Square (*χ* ^2^)

The selection of the best drying model is based on statistical values and mathematical models. The coefficient of determination (*R* ^2^) is used to select the best equation that describes the experimental data of the drying process. In addition to the coefficient of determination, the chi‐square (*χ* ^2^) value has been used to determine the degree of compatibility with the experimental models. The rate constant (*k*) of the process is determined by a nonlinear regression equation based on experimental values derived from the graph of moisture content and time (*t*) (Vu, Pham, et al. 2025).

### 2.5 Statistical Analysis and Data

The data were calculated using Microsoft Excel software (Redmond, WA, USA). Origin Pro 9.0 software, version 90E (OriginLab, Roundhouse Plaza Northampton, USA), was used to determine statistical parameters and kinetic models. Statgraphics Centurion XV, version 15.1.02, was used to indicate statistical differences between conditions (Nhi et al. 2020).

## 3 Result and Discussion

### 3.1 Influence of Storage Temperature and Duration on the Moisture Content of Mango Powder

Moisture content is an important factor in protecting and maintaining the function of food (Gaikwad et al. 2019). High moisture content in products can promote the growth of microorganisms. At the same time, products with higher moisture absorption are more likely to undergo changes in texture and properties, leading to the degradation of essential components, thus reducing shelf life (Ma et al. 2020). In real‐world scenarios, powdered products are often subject to varying storage environments—from supermarkets and local stores to harsh conditions during transit or in climate‐sensitive regions.

**FIGURE 1:**
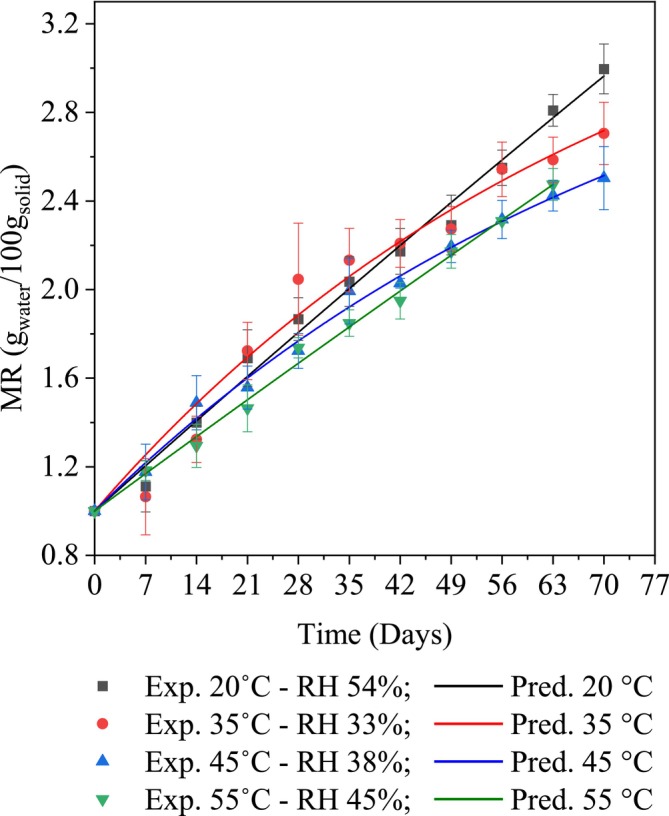
The Vu kinetic model describes the moisture absorption mechanism under different storage conditions.

Storage temperatures ranging from 20°C to 55°C with environmental RH from 33% to 54% were considered to represent most of the practical storage conditions for powdered products (Dai et al. 2022). The results indicated a significant effect of temperature, RH, and storage time on the moisture absorption process of mango powder (*p* < 0.05) (Figure 1). In general, moisture absorption was primarily driven by the relative humidity, with temperature acting as a catalytic factor. The results found that after the first 21 days of storage at 35°C—RH 33%, the moisture content in mango powder increased very quickly from 1.00 to 1.72 g_water_/100 g_solid_, corresponding to an increase of approximately 1.62%, but the rate slowed in subsequent days, with only an additional 1.04% absorbed beyond the initial level. However, under conditions with higher environmental RH such as 20°C—RH 54%, 45°C—RH 38%, and 55°C—RH 45%, the moisture absorption process also occurred more quickly and continuously, with 1.52%, 1.33%, and 1.24% absorbed after the first 40 days, respectively, and then slowed down in the following days. Indeed, a previous report also indicated that storage of powdered products at higher environmental humidity resulted in faster and continuous moisture absorption (Bovi et al. 2018). The transition between the two phases of fast and slow moisture absorption is represented by the inflection point of the moisture absorption curve. The increase at 20°C occurred quickly from 0 to 40 days, with the rapid increase in moisture content due to the low environmental temperature and high relative humidity, which created favorable conditions for significant moisture absorption. After 40 days, the rate of absorption showed signs of slowing down. On the other hand, at 35°C—RH 33%, moisture absorption was quite good in the initial phase from 0 to 40 days at an average temperature. However, high temperatures and low RH cause a low steam application effect, leading to a lower moisture absorption capacity. Although the MR increases more slowly at 20°C, it still maintains a steady increase. For 45°C—RH 38% and 55°C—RH 45%, the MR increase rate is relatively slow in the initial stage, and from Day 56 onward, the absorption rate gradually decreases. The high temperature generates steam, causing reverse evaporation, and the structure may be damaged, reducing the ability to retain moisture.

Additionally, the storage process from Day 7 to Day 56 at 55°C showed lower moisture content compared to storage at 45°C. However, after Day 63 at 55°C, the moisture content began to increase more rapidly. This indicated an overlap on Day 56, mainly influenced by temperature. On the other hand, the moisture content of the sample increased slowly during storage at 45°C–55°C. This could be due to the impact of high temperatures, which increased the moisture separation capacity of the material. Furthermore, the moisture absorption curves at storage conditions of 20°C—RH 54%, 45°C—RH 38%, and 55°C—RH 45% suggested that the samples had not yet reached equilibrium moisture content.

Moreover, the moisture absorption process of mango powder exhibited a trend similar to the high moisture absorption capacity of spray‐dried melon powder when the relative humidity of the environment increased from 50% to 90% (Tan et al. 2021). Moisture absorption in fennel seeds showed two distinct phases: an initial rapid phase (~500 min), followed by a slower phase lasting up to 1500 min under high humidity (Bidkhori and Mohammadpour Karizaki 2022). A previous report also indicated a positive correlation between environmental relative humidity and the moisture content of fruits, with temperature having little or no significant effect on this correlation (Pashazadeh 2020).

On the other hand, common kinetic models have often been used to describe the moisture absorption mechanism of various materials, including the first‐order model for banana moisture absorption (Yan et al. 2008), the Peleg model for spice powder moisture absorption (Niamnuy et al. 2008; Thuy et al. 2024), the Weibull model for fresh mushroom (*Agaricus bisporous*) moisture absorption (Mahajan et al. 2008), and the Vu model for soursop tea powder moisture absorption (Vu et al. 2024). These models were then tested based on their fit with the experimental moisture content data of mango powder under different storage temperature conditions. Experimental results indicated that the Vu model was among the most accurate for predicting the moisture absorption of mango powder during storage at temperature conditions from 20°C to 55°C (*R* ^2^ > 0.99). Based on the statistical results, experimental kinetic models based on the Vu model were developed for each optimal mango powder storage condition, showing the highest *R* ^2^ values (0.97137–0.99513), the lowest *χ* ^2^ (0.00218–0.01751), and the smallest RMSE (0.04668–0.13231) (Table 1).

**TABLE 1:** Kinetic parameters describing moisture absorption during storage.

No.	Models	Parameters	Temperatures (°C)			
			20	35	45	55
1	First‐order	*χ* ^2^	0.03008	0.07223	0.04072	0.01172
		RMSE	0.17343	0.26875	0.20180	0.10824
		*R* ^2^	0.93054	0.80313	0.84053	0.95290
2	Peleg	*χ* ^2^	0.00385	0.01200	0.00194	0.00136
		RMSE	0.06207	0.10954	0.04402	0.03690
		*R* ^2^	0.99199	0.97056	0.99317	0.99513
3	Weibull	*χ* ^2^	0.00578	0.01751	0.00287	0.00218
		RMSE	0.07603	0.13231	0.05355	0.04668
		*R* ^2^	0.99199	0.97137	0.99326	0.99513
4	Vu	*χ* ^2^	0.00385	0.01167	0.00191	0.00136
		RMSE	0.06208	0.10803	0.03373	0.03691
		*R* ^2^	0.99211	0.97371	0.99526	0.99617

Additionally, statistical analysis results showed that the rate constants (*k*_1_) and (*k*_2_) at storage conditions with temperature and relative humidity of 20°C—RH 54%, 35°C—RH 33%, 45°C—RH 38%, and 55°C—RH 45% were 29.16506, 7.58561, 6.43637, 34.27212 (*k*_1_) and 7.19152, 3.59559, 3.35314, and 7.74978 (*k*_2_); the kinetic parameters *β* of the Vu model were 786.78094, 73.66351, 79.64584, and 939.38403, respectively (Table 2). Based on the model parameters and rate constants, the mathematical equations describing the moisture absorption behavior in mango powder during storage at different temperature and relative humidity conditions at 20°C, 35°C, 45°C, and 55°C are presented as follows (Equations (8), (9), (10), (11)):

(8) $$20^{{}^{\circ}}C:{\mathrm{MR}}_{t}=24.03\times \left[1–\exp\;\left(-t/786.78\right)\right]$$

(9) $$35^{{}^{\circ}}C:{\mathrm{MR}}_{t}=3.78\times \left[1–\exp\;\left(-t/73.66\right)\right]$$

(10) $$45^{{}^{\circ}}C:{\mathrm{MR}}_{t}=3.18\times \left[1–\exp\left(-t/79.65\right)\right]$$

(11) $$55^{{}^{\circ}}C:{\mathrm{MR}}_{t}=27.72\times \left[1–\exp\;\left(-t/939.38\right)\right]$$

**TABLE 2:** Moisture absorption rate constants and the parameters of the Vu model.

No.	Parameters	Temperatures (°C)			
		20	35	45	55
1	*n*_1_	1.00004	1.00004	1.00028	1.00127
2	*n*_2_	−2.60386	−0.40473	−0.46135	−1.90135
3	*k*_1_	29.16506	7.58561	6.43637	34.27212
4	*k*_2_	7.19152	3.59559	3.35314	7.74978
5	*β*	786.78094	73.66351	79.64584	939.38403

The Vu model accurately describes the moisture absorption process in mango powder when stored under the corresponding conditions (*R* ^2^ > 0.99). The models accurately predict the specific time for significant moisture content changes under storage conditions. This serves as the basis for controlling product quality based on moisture criteria during storage, allowing for easy prediction of the product's shelf life.

### 3.2 Influence of Storage Temperature and Duration on the Total Flavonoid Content of Mango Powder

Environmental factors, particularly temperature and humidity, can accelerate undesirable chemical reactions that degrade bioactive compounds during storage.

**FIGURE 2:**
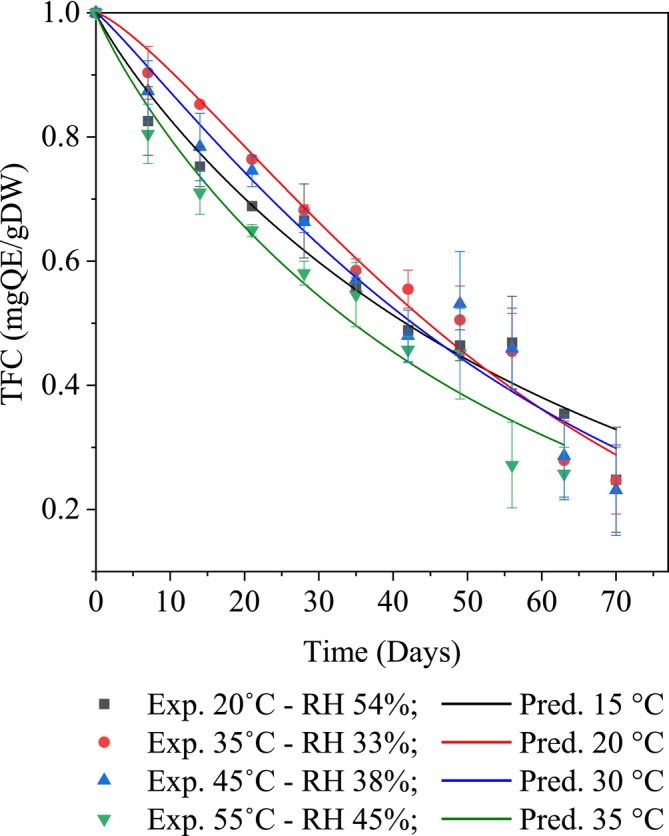
The Weibull kinetic model describes the degradation of flavonoids under different storage conditions.

Flavonoids are natural polyphenolic compounds found in fruits, vegetables, tea, and wine, known for their antioxidant, anti‐inflammatory, and cancer‐preventive properties (Griffiths et al. 2016). Structurally, flavonoids are a subclass of polyphenols, with a structure containing multiple benzene rings and hydroxyl groups (–OH), which give them strong antioxidant properties (Olszowy 2019). Preserving flavonoids is therefore crucial for maintaining the functional and nutritional quality of food products. Experimental results demonstrated a gradual decline in total flavonoid content during storage under varying temperature and relative humidity conditions (Figure 2). Within the first 35 days, significant degradation of flavonoids (*p* < 0.05) was observed at all four temperature levels (20°C–55°C), primarily due to elevated oxygen levels inside the packaging. As temperature increased, the degradation rate accelerated, reflecting the thermal sensitivity of flavonoid structures, particularly their ring and glycosidic linkages (Lin and Xiao 2024). Furthermore, ANOVA results indicated that both storage temperature and duration had a significant effect on flavonoid content (*p* < 0.05). However, the difference in flavonoid degradation between 35°C and 45°C was not statistically significant (*p* > 0.05). Flavonoid degradation at 35°C and 45°C proceeded more slowly than at 20°C and 55°C. This may be attributed to the intermediate temperatures and relatively low relative humidity, which together created conditions less conducive to flavonoid degradation. After 50 days of storage, the degradation processes converged to a similar concentration, corresponding to approximately 40% of the initial content. Beyond this point, the degradation rate plateaued, suggesting the presence of a threshold or equilibrium level, below which further degradation was limited. This stabilization may be due to the resistance of remaining flavonoid structures to oxidative breakdown. Additionally, the interaction between temperature and relative humidity may have contributed to this observed plateau, resulting in what appeared to be a “reversal” or slowing of the degradation trend (Pérez‐Vicente et al. 2002).

Furthermore, the degradation of flavonoids during storage at 55°C occurred continuously, primarily due to the combination of high temperature and relative humidity, which strongly accelerated the degradation process. A previous study also indicated that elevated temperatures could disrupt cell membranes, thereby releasing oxidative enzymes that promote the breakdown of antioxidant compounds in food (Do et al. 2024). Similarly, a study on soursop tea reported that condensation at low temperatures increased the moisture content. Specifically, storage at 20°C under high humidity (RH = 54%) led to greater moisture absorption and further promoted degradation over extended storage periods (Vu et al. 2024).

The degradation of flavonoids in freeze‐dried mango powder was modeled using zero‐order, first‐order, and Weibull kinetic equations across four temperature levels ranging 20°C–55°C (Table 3). Among these, Weibull model provided the best fit for describing total flavonoid content degradation, based on statistical indicators such as the lowest *χ* ^2^ values (0.00151–0.00264), the smallest RMSE values (0.03887–0.05141), and the highest *R* ^2^ values (0.95814–0.97753). These results indicated that the Weibull model accurately captured the low variability in the experimental data and delivered high predictive performance for the experimental data.

**TABLE 3:** Kinetic parameters describing the degradation of TFC during storage.

No.	Models	Parameters	Temperatures (°C)			
			20	35	45	55
1	Zero‐order	*χ* ^2^	0.00405	0.00085	0.00242	0.00489
		RMSE	0.06365	0.02916	0.04915	0.06995
		*R* ^2^	0.91548	0.98595	0.95748	0.90825
2	First‐order	*χ* ^2^	0.00176	0.00268	0.00262	0.00209
		RMSE	0.04200	0.05175	0.05115	0.04568
		*R* ^2^	0.96320	0.95573	0.95395	0.96087
3	Weibull	*χ* ^2^	0.00180	0.00151	0.00264	0.00213
		RMSE	0.04242	0.03887	0.05141	0.04618
		*R* ^2^	0.96621	0.97753	0.95814	0.96445

Additionally, the estimated rate constants (*k*) under various temperature and relative humidity of 20°C—RH 54%, 35°C—RH 33%, 45°C—RH 38%, and 55°C—RH 45% were 0.01605, 0.01689, 0.01692, and 0.01925, respectively (Table 4). Based on these statistical outcomes and degradation rate constants, mathematical models were developed to describe the degradation mechanisms of flavonoid compounds in mango powder during storage under different environmental conditions of temperature and relative humidity (Equations (12), (13), (14), (15)).

(12) $$20^{{}^{\circ}}C:{\mathrm{TFC}}_{t}=C_{0}\times \exp\;\left[-{\left(0.01605\times t\right)}^{0.91198}\right]$$

(13) $$35^{{}^{\circ}}C:{\mathrm{TFC}}_{t}=C_{0}\times \exp\;\left[-{\left(0.01689\times t\right)}^{1.30618}\right]$$

(14) $$45^{{}^{\circ}}C:{\mathrm{TFC}}_{t}=C_{0}\times \exp\;\left[-{\left(0.01692\times t\right)}^{1.12160}\right]$$

(15) $$55^{{}^{\circ}}C:{\mathrm{TFC}}_{t}=C_{0}\times \exp\;\left[-{\left(0.01925\times t\right)}^{0.90255}\right]$$

**TABLE 4:** Degradation rate constants of flavonoids and the parameters of the Weibull model.

No.	Temperatures (°C)	Parameters	
		*k*_1_ (day^−1^)	*n*
1	20	0.01605	0.91198
2	35	0.01689	1.30618
3	45	0.01692	1.12160
4	55	0.01925	0.90255

These models accurately described the degradation behavior of flavonoids in mango powder under the tested storage conditions (*R* ^2^ > 0.95). They also predicted the time required for flavonoid degradation to reach a significant threshold under each storage condition. These findings provide a basis for optimizing storage conditions and estimating the shelf life of flavonoid in the product.

### 3.3 Influence of Storage Temperature and Duration on the Vitamin C of Mango Powder

Temperature, humidity, and external stressors such as light and oxygen can trigger oxidative reactions, leading to the degradation of vitamin C and other sensitive nutrients.

**FIGURE 3:**
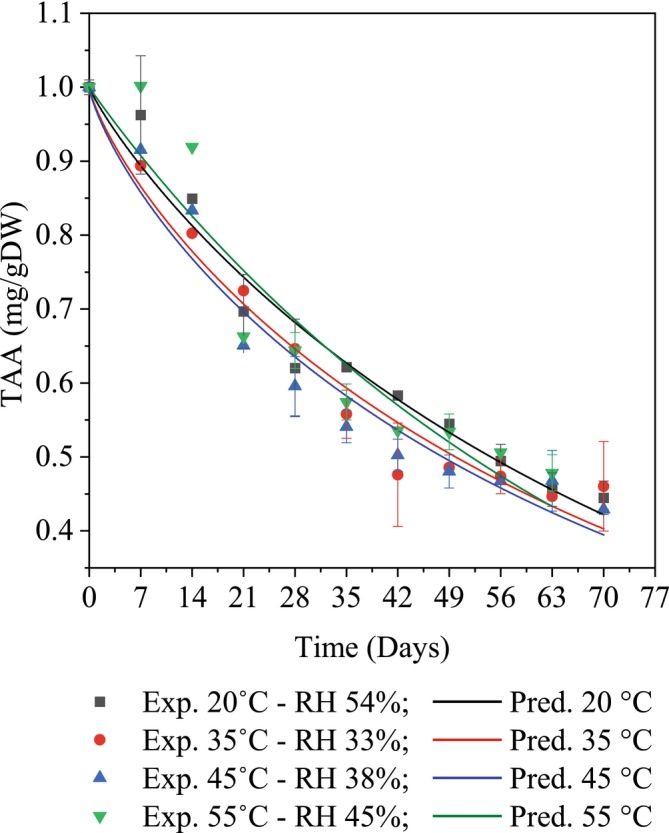
The Weibull kinetic model describes the degradation of TAA under different storage conditions.

Vitamin C, also known as ascorbic acid, is a potent antioxidant essential for various biological processes, including immune function, iron absorption, and collagen synthesis (Mumtaz et al. 2021). Moreover, it helps regenerate and protect other antioxidant compounds (Mumtaz et al. 2021). Statistical analysis revealed a significant overall decline in vitamin C content during storage across all tested conditions (*p* < 0.05), with the most pronounced losses occurring between Days 7 and 35 (Figure 3). However, when comparing the individual effects of the four storage temperatures, no statistically significant differences were observed (*p* > 0.05), suggesting that temperature alone did not distinctly influence vitamin C degradation. Instead, the degradation appeared to be driven by cumulative exposure to oxygen, humidity, and light (Lešková and Morochovičová 2006). Besides, It is possible that the opposing influences of temperature and relative humidity contributed to a stabilizing effect (Jutkus et al. 2015). At high temperatures, although thermal degradation is more likely, the low relative humidity might have limited moisture‐related vitamin C breakdown. Conversely, at lower temperatures with higher RH, oxidative degradation could have been more prominent due to greater moisture availability, despite reduced thermal stress. These counteracting factors may have resulted in comparable overall degradation levels across the tested storage conditions (Do et al. 2024). Besides, after 70 days of storage, the remaining vitamin C content was approximately 0.043 ± 0.003 mg/gDW, with minimal variation between the tested conditions. A previous study also reported no significant differences in the effects of thermal treatment ranging from 50°C to 70°C on convectively dried mango powder (0.13%–0.14%) (Agustini 2018). A similar report on the thermal impact on vitamin C in cashew apple powder at 55°C–61°C also showed no statistically significant differences (106–110 mg/100 g) (Prakoso and Mubarok 2021).

Vitamin C degradation in mango powder was modeled using zero‐order, first‐order, and Weibull models. Among these, the Weibull model best described the TAA degradation data, with *χ* ^2^ values (0.00131–0.00428), RMSE values (0.03622–0.06541), and *R* ^2^ values (0.91188–0.96677) (Table 5). These results confirmed that the Weibull model effectively captured the low variability in experimental observations and offered high predictive accuracy, enabling reliable modeling of vitamin C degradation in the product.

**TABLE 5:** Kinetic parameters describing the degradation of TAA during storage.

No.	Models	Parameters	Temperatures (°C)			
			20	35	45	55
1	Zero‐order	*χ* ^2^	0.00416	0.00658	0.00814	0.00600
		RMSE	0.06447	0.08110	0.09024	0.07743
		*R* ^2^	0.89115	0.82778	0.79687	0.86110
2	First‐order	*χ* ^2^	0.00150	0.00198	0.00277	0.00381
		RMSE	0.03870	0.04446	0.05267	0.06172
		*R* ^2^	0.96078	0.94824	0.93081	0.91175
3	Weibull	*χ* ^2^	0.00141	0.00131	0.00193	0.00428
		RMSE	0.03754	0.03622	0.04395	0.06541
		*R* ^2^	0.96677	0.96907	0.95663	0.91188

Moreover, statistical analysis results showed the rate constant (*k*) under storage conditions at 20°C—RH 54%, 35°C—RH 33%, 45°C—RH 38%, and 55°C—RH 45% were 0.01210, 0.01269, 0.01302, and 0.01324, respectively, with the rate increasing slightly—by approximately 1.01–1.05 times—from 20°C to 45°C (Table 6). Notably, from 45°C to 55°C, the increase was minimal (only 1.01 times), suggesting a plateau effect at higher temperatures (Thuy et al. 2024). Based on the model parameters and degradation rate constants, the mathematical equations describing the degradation mechanisms of vitamin C in mango powder during storage at different environmental temperature/relative humidity levels, with model parameters *n* at 20°C, 35°C, 45°C, and 55°C, were 0.88657, 0.80092, 0.78334, and 0.98129, respectively, and were presented as follows (Equations (16), (17), (18), (19)):

(16) $$20^{{}^{\circ}}C:{\mathrm{TAA}}_{t}=C_{0}\times \exp\;\left[-{\left(0.01210\times t\right)}^{0.88657}\right]$$

(17) $$35^{{}^{\circ}}C:{\mathrm{TAA}}_{t}=C_{0}\times \exp\;\left[-{\left(0.01269\times t\right)}^{0.80092}\right]$$

(18) $$45^{{}^{\circ}}C:{\mathrm{TAA}}_{t}=C_{0}\times \exp\;\left[-{\left(0.01302\times t\right)}^{0.78334}\right]$$

(19) $$55^{{}^{\circ}}C:{\mathrm{TAA}}_{t}=C_{0}\times \exp\;\left[-{\left(0.01324\times t\right)}^{0.98129}\right]$$

**TABLE 6:** Degradation rate constants of antioxidant activities and the parameters of the Weibull model.

No.	Temperatures (°C)	Parameters	
		*k*_1_ (day^−1^)	*n*
1	20	0.01210	0.88657
2	35	0.01269	0.80092
3	45	0.01302	0.78334
4	55	0.01324	0.98129

These models accurately described the degradation of vitamin C in mango powder under the tested storage conditions (*R* ^2^ > 0.91). They also predicted the time required for vitamin C degradation to reach a significant threshold for each storage condition. These findings provide a basis for optimizing storage conditions and estimating the shelf life of vitamin C in the product.

### 3.4 Influence of Storage Temperature and Duration on the DPPH and ABTS Antioxidant Activities of Mango Powder

The variation in antioxidant activity is primarily dependent on bioactive components such as polyphenols, flavonoids, vitamin C, and other components.

**FIGURE 4:**
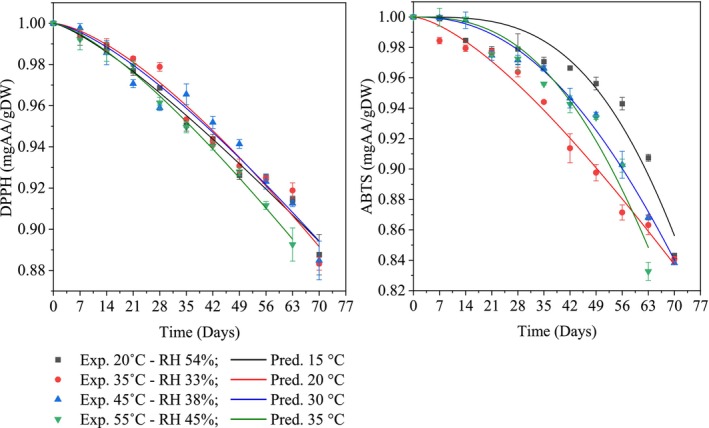
The Weibull kinetic model describes the degradation of antioxidant activities (DPPH and ABTS) under different storage conditions.

DPPH (2,2‐Diphenyl‐1‐picrylhydrazyl) and ABTS (2,2′‐Azino‐bis (3‐ethylbenzothiazoline‐6‐sulfonic acid)) are free radicals and precursors of free radicals commonly used to measure the antioxidant activity of food products. A total of three kinetic models were developed to describe the degradation of DPPH and ABTS antioxidant activity during storage, including the zero‐order model, first‐order model, and Weibull model. The storage process led to a gradual decrease in both DPPH and ABTS antioxidant activities, which was significantly accelerated at higher temperatures (Figure 4). The degradation was largely attributed to the reduced stability and gradual breakdown of key antioxidant compounds, especially flavonoids. As the temperature increased from 20°C to 45°C, decomposition reactions were facilitated. Notably, the decline in DPPH antioxidant activity was most pronounced at 55°C. The higher the temperature, the more the biological reactions are activated and accelerated, causing bonds in bioactive components to become unstable and easily break. Consequently, the antioxidant activity significantly declined (Thuy et al. 2024). A previous study also reported a positive correlation between flavonoid content and antioxidant activity (DPPH and ABTS) in 13 banana cultivars (Vu, Hang, et al. 2025). In agreement with this finding, a positive correlation between flavonoid content and DPPH antioxidant activity was also reported in various Malaysian honey samples, with *R* ^2^ values exceeding 0.88 (Zawawi et al. 2021). On the other hand, higher storage temperatures were shown to have a significantly negative impact on the antioxidant activity (FRAP) of roselle‐fruit juice (Mgaya‐Kilima et al. 2014).

Furthermore, developing an appropriate model to describe the degradation mechanism of DPPH antioxidant content in fruit powder products led to the conclusion that the Weibull model is the most suitable for predicting DPPH antioxidant content during storage in mango powder. The rate constants (*k*) results demonstrated that increasing the storage temperature from 20°C to 55°C accelerated the degradation rate. Indeed, the degradation rate constant increased from 0.00262 to 0.00325. On the other hand, for ABTS antioxidant activity, after 42 days of storage at four temperature levels from 20°C to 55°C, ABTS antioxidant activity rapidly decreased over time. This rapid decline may be due to exposure to oxygen in the packaging, leading to decomposition reactions of bioactive compounds. However, different storage temperatures had varying effects on ABTS antioxidant activity. Storage at 35°C showed a sharp decrease in ABTS antioxidant activity in mango powder, with approximately a 10% decrease after 47 days of storage. Storage at 20°C was found to better preserve ABTS antioxidant activity, showing only a 5% reduction after 47 days. Furthermore, the differences in DPPH and ABTS antioxidant activity could be due to the different mechanisms of free radical activity. However, the overall antioxidant activity of mango powder after 70 days of storage showed a reduction of about 15%–25% in antioxidant effectiveness. This low degradation could be attributed to the inverse relationship between the components of antioxidants such as polyphenols, flavonoids, and vitamin C (Nguyễn et al. 2023).

On the other hand, the degradation rate constant (*k*) of DPPH and ABTS antioxidant activity under different storage conditions was considered, and the Weibull model was selected as the most suitable model. The results showed that the degradation rate of DPPH antioxidant activity (*k*) ranged from 0.00262 to 0.00335 day^−1^. Specifically, high temperatures (35°C) or humid environments combined with low temperatures (20°C—RH 54%) accelerated the degradation process of antioxidant activity. On the other hand, the degradation rate of ABTS antioxidant activity (*k*) was found to range from 0.00448 to 0.00797 day^−1^. A comparison of the degradation rates of DPPH and ABTS antioxidant activity showed that the DPPH activity was less neutralized by bioactive components compared to the ABTS method. However, the difference in affinity between free radicals and antioxidants also caused differences in the overall antioxidant activity of mango powder.

Statistical analysis revealed that the Weibull model provided the best fit for describing the degradation of both DPPH and ABTS antioxidant activities (Table 7). Specifically, *χ* ^2^ values ranged from 0 to 0.00005 for DPPH and from 0.00004 to 0.00016 for ABTS. Corresponding RMSE values were 0.00267–0.00694 for DPPH and 0.00649–0.01291 for ABTS. Additionally, the model achieved high *R* ^2^ values: 0.96685–0.98527 for DPPH and 0.92941–0.98737 for ABTS. These results confirm that the Weibull model not only captured the low variability in the experimental data but also demonstrated high predictive accuracy. Therefore, it was selected as the most appropriate model for predicting the degradation behavior of antioxidant activities in mango powder during storage. The model parameter (*n*), which reflects the shape of the degradation curve, varied across storage temperatures (20°C, 35°C, 45°C, and 55°C), with values for DPPH of 1.29029, 1.49250, 1.40779, and 1.38785, respectively, and for ABTS of 3.28766, 1.48891, 2.26967, and 2.61949, respectively (Table 8). Based on these parameters, the corresponding Weibull kinetic equations describing the degradation of DPPH and ABTS antioxidant activities under different storage conditions were subsequently established (Equations (20), (21), (22), (23), (24), (25), (26), (27)).

(20) $$20^{{}^{\circ}}C:{\text{DPPH}}_{t}=C_{0}\times \exp\;\left[-{\left(0.00262\times t\right)}^{1.29029}\right]$$

(21) $$35^{{}^{\circ}}C:{\text{DPPH}}_{t}=C_{0}\times \exp\;\left[-{\left(0.00335\times t\right)}^{1.49250}\right]$$

(22) $$45^{{}^{\circ}}C:{\text{DPPH}}_{t}=C_{0}\times \exp\;\left[-{\left(0.00301\times t\right)}^{1.40779}\right]$$

(23) $$55^{{}^{\circ}}C:{\text{DPPH}}_{t}=C_{0}\times \exp\;\left[-{\left(0.00325\times t\right)}^{1.38785}\right]$$

(24) $$20^{{}^{\circ}}C:{\text{ABTS}}_{t}=C_{0}\times \exp\;\left[-{\left(0.00811\times t\right)}^{3.28766}\right]$$

(25) $$35^{{}^{\circ}}C:{\text{ABTS}}_{t}=C_{0}\times \exp\;\left[-{\left(0.00448\times t\right)}^{1.48891}\right]$$

(26) $$45^{{}^{\circ}}C:{\text{ABTS}}_{t}=C_{0}\times \exp\;\left[-{\left(0.00661\times t\right)}^{2.26967}\right]$$

(27) $$55^{{}^{\circ}}C:{\text{ABTS}}_{t}=C_{0}\times \exp\;\left[-{\left(0.00797\times t\right)}^{2.61949}\right]$$

**TABLE 7:** Kinetic parameters describing the degradation of antioxidant activities (DPPH and ABTS) during storage.

No.	Indicators	Models	Parameters	Temperatures (°C)			
				20	35	45	55
1	DPPH antioxidant activity	Zero‐order	*χ* ^2^	0.00004	0.0001	0.00008	0.00004
			RMSE	0.00646	0.00999	0.00894	0.00661
			*R* ^2^	0.96829	0.92997	0.93889	0.96665
		First‐order	*χ* ^2^	0.00005	0.00011	0.00009	0.00005
			RMSE	0.00694	0.01047	0.00939	0.00717
			*R* ^2^	0.96347	0.92317	0.93257	0.96073
		Weibull	*χ* ^2^	0.00002	0.00004	0.00005	0.00000
			RMSE	0.00464	0.00656	0.00694	0.00267
			*R* ^2^	0.98527	0.97282	0.96685	0.99515
2	ABTS antioxidant activity	Zero‐order	*χ* ^2^	0.00061	0.00017	0.00048	0.00061
			RMSE	0.02465	0.01286	0.02196	0.02470
			*R* ^2^	0.71414	0.94678	0.83923	0.77922
		First‐order	*χ* ^2^	0.00063	0.00019	0.00052	0.00064
			RMSE	0.02513	0.01406	0.02289	0.02540
			*R* ^2^	0.70293	0.93633	0.82532	0.76654
		Weibull	*χ* ^2^	0.00016	0.00004	0.00004	0.00014
			RMSE	0.01291	0.00682	0.00649	0.01197
			*R* ^2^	0.92941	0.98654	0.98737	0.95392

**TABLE 8:** Degradation rate constants of antioxidant activities (DPPH and ABTS) and parameters of the Weibull kinetic model.

No.	Indicators (antioxidant activities)	Temperatures (°C)	Parameters	
			*k*_1_ (day^−1^)	*n*
1	DPPH	20	0.00262	1.29029
		35	0.00335	1.49250
		45	0.00301	1.40779
		55	0.00325	1.38785
2	ABTS	20	0.00811	3.28766
		35	0.00448	1.48891
		45	0.00661	2.26967
		55	0.00797	2.61949

The models accurately predict the specific time for significant degradation of DPPH and ABTS antioxidant activity under storage conditions. This serves as the basis for controlling antioxidant capacity and estimating the shelf life of mango powder in order to maintain the product's high antioxidant activity.

### 3.5 Applications of Mathematical Models to Predict the Shelf Life of Mango Powder

Shelf life refers to the period during which a food product maintains its nutritional quality and remains safe for consumption under the storage conditions indicated on its packaging. Accurately determining the shelf life is crucial for product classification, distribution planning and especially export strategies, where extended shelf life is often a key requirement to meet international quality standards. The selected evaluation criteria served as a basis for determining shelf life, with each criterion yielding a different predicted duration. Accordingly, mathematical models were developed to predict shelf life under specific storage conditions. The results indicated that the maximum shelf life of mango powder, based on polyphenol content, is approximately 128.65 days, with an 80% reduction in content at 20°C—RH 54% (~4.3 months) (Table 9). Total flavonoid content is best preserved at 20°C—RH 54%, with a shelf life of 104.99 days (~3.5 months). Additionally, Vitamin C exhibited the greatest stability at 35°C—RH 33%, with a shelf life of 142.75 days (~4.8 months). Although bioactive components decrease over time, DPPH antioxidant activity can last up to 551.92 days at 20°C—RH 54% (~1.5 years), and ABTS antioxidant activity can last up to 307.28 days at 35°C—RH 33% (~10.2 months). This can be attributed to the formation of new stable antioxidant compounds as bioactive components degrade (Munteanu and Apetrei 2021). Additionally, antioxidant compounds can interact synergistically or additively when working together (Jalali et al. 2022).

**TABLE 9:** Shelf life (days) of freeze‐dried Tu Quy mango powder depending on different storage conditions and quality criteria.

No.	Indicators	Temperatures (°C)			
		20	35	45	55
1	Moisture content^a^	∞	∞	∞	∞
2	Total flavonoids content^b^	104.99	85.23	90.34	88.02
3	Vitamin C^b^	141.36	142.75	141.00	122.67
4	DPPH antioxidant activity^b^	551.92	410.61	465.84	433.54
5	ABTS antioxidant activity^b^	142.51	307.28	186.58	150.47

^a^ Shelf life is calculated based on a 5% increase in moisture content.

^b^ Shelf life is determined based on an 80% decrease in bioactive component content compared to the initial level.

## 4 Conclusion

This study investigated the drying and storage processes of mango powder ( *Mangifera indica* L.), focusing on changes in its bioactive components and antioxidant capacity. The results indicated that mango powder exhibits strong moisture absorption, particularly under high relative humidity conditions. Among the tested kinetic models, the first‐order, Weibull, and Vu models effectively described the degradation processes and can be applied to similar dried products. Specifically, the first‐order and Weibull models were found to be particularly suitable for predicting changes in flavonoid content, which significantly degraded during storage depending on environmental temperature and humidity. Vitamin C content significantly decreased during storage, especially under high‐temperature conditions. The Weibull model also demonstrated high suitability for predicting the degradation of Vitamin C. The antioxidant activity of mango powder decreased over storage time, but it retained 80% of its initial antioxidant activity. The research results provided valuable insights into the mechanisms and rates of changes in bioactive components, and the models describing the storage process of mango powder. This not only helps optimize the production and storage processes but also provides a scientific basis for developing automated systems to control the storage process of mango powder and similar products.

## Author Contributions

**Ngoc Duc Vu:** conceptualization (lead), data curation (lead), formal analysis (lead), investigation (lead), methodology (lead), project administration (lead), resources (lead), software (lead), supervision (lead), validation (lead), visualization (lead), writing – original draft (lead), writing – review and editing (lead). **Trinh Thi Nhu Hang Nguyen:** formal analysis (equal), software (equal). **Thi Hoang Nhi Dang:** formal analysis (equal), software (equal). **Binh An Pham:** formal analysis (equal), project administration (equal), software (equal).

## Conflicts of Interest

The authors declare no conflicts of interest.

## Data Availability

All the data are available within this manuscript.
